# The impact of an integrated diabetes and kidney service on patients, primary and specialist health professionals in Australia: A qualitative study

**DOI:** 10.1371/journal.pone.0219685

**Published:** 2019-07-15

**Authors:** Edward Zimbudzi, Clement Lo, Tracy Robinson, Sanjeeva Ranasinha, Helena J. Teede, Tim Usherwood, Kevan R. Polkinghorne, Peter G. Kerr, Gregory Fulcher, Martin Gallagher, Stephen Jan, Alan Cass, Rowan Walker, Grant Russell, Greg Johnson, Sophia Zoungas

**Affiliations:** 1 School of Public Health and Preventive Medicine, Monash University, Melbourne, Australia; 2 Department of Nephrology, Monash Health, Melbourne, Australia; 3 Diabetes and Vascular Medicine Unit, Monash Health, Melbourne, Australia; 4 The George Institute for Global Health, University of New South Wales, Sydney, Australia; 5 Department of General Practice, Sydney Medical School, University of Sydney, Sydney, Australia; 6 School of Clinical Sciences, Monash University, Melbourne, Australia; 7 Department of Diabetes, Endocrinology & Metabolism, Royal North Shore Hospital, University of Sydney, Sydney, Australia; 8 Northern Clinical School, University of Sydney, Sydney, Australia; 9 Concord Clinical School, University of Sydney, Sydney, Australia; 10 Sydney Medical School, University of Sydney, Sydney, Australia; 11 Menzies School of Health Research, Darwin, Australia; 12 Department of Renal Medicine, Alfred Health, Melbourne, Australia; 13 School of Primary Health Care, Monash University, Melbourne, Australia; 14 Diabetes Australia, Canberra, Australia; Swinburne University of Technology, AUSTRALIA

## Abstract

**Background:**

To address guideline-practice gaps and improve management of patients with both diabetes and chronic kidney disease (CKD), we involved patients, health professionals and patient advocacy groups in the co-design and implementation of an integrated diabetes-kidney service.

**Objective:**

In this study, we explored the experiences of patients and health-care providers, within this integrated diabetes and kidney service.

**Methods:**

5 focus groups and 2 semi-structured interviews were conducted amongst attending patients, referring primary health professionals, and attending specialist health professionals. Maximal variation sampling was used for both patients and referring primary health professionals to ensure an equal representation of males and females, and patients of different CKD stages. All discussions were audiotaped and transcribed verbatim, before being thematically analysed independently by 2 researchers.

**Results:**

The mean age (SD) for specialist health professionals, primary care professionals and patients who participated was 45 (11), 44 (15) and 68 (5) years with men being 50%, 80% and 76% of the participants respectively. Key strengths of the diabetes and kidney service were noted to be better integration of care and a perception of improved health and management of health. Whilst some aspects of access such as time between referral and initial appointment and having fewer appointments improved, other aspects such as in-clinic waiting times and parking remained problematic. Specialist health professionals noted that health professional education could be improved. Patient self-management was also noted by to be an issue with some patients requesting more information and some health professionals expressing difficulty in empowering some patients.

**Conclusions:**

Health professionals and patients reported that a co-designed integrated diabetes kidney service improved integration of care and improved health and management of health. However, some aspects of the process of care, health professional education and patient self-management remained challenging.

## Introduction

Multimorbidity, the co-occurrence of multiple chronic conditions in an individual, is increasing as our global population is living longer but with more chronic, non-communicable diseases [1, 2]. Patients with multimorbidity often have complex health needs which transcend the traditional disease-orientated specialist service approach and this may lead to fragmentation of and suboptimal care [2]. For example, patients with co-morbid diabetes and chronic kidney disease (CKD) often do not receive monitoring consistent with recommended standards of care such as regular HbA1c monitoring or screening for albuminuria, and many do not attain recommended glycaemic and blood pressure targets [3–9].

There is a clear need to integrate across specialty health services especially for patients with complex health-care needs such as those with both diabetes and CKD. Thus, we co-designed an integrated model of care for patients with both diabetes and CKD involving patients, health professionals and also consumer advocacy organisations (Diabetes Australia and Kidney Health Australia). This was informed by findings from a large multi-site formative evaluation of the barriers and enablers of optimal health-care for diabetes and CKD, and the needs of patients, carers, and their health professionals published [9–12]. The model of care has been described in detail previously [13], but key components of the integrated service are:

1. The primary health professional (general practitioner GP) remains the patient’s primary care giver and coordinator of care
2. Specialist services are provided by an integrated diabetes-kidney service consisting of diabetes and renal physicians, nurse practitioners and a dietitian.
3. Care is person-centred, focusing on facilitating self-management of disease. A key component of this is a diabetes-kidney care plan that is given to the patient after each visit (to reinforce the management plan agreed upon by the service and the patient).
4. Care is structured according to an electronic history proforma.
5. To facilitate interdisciplinary management of each patient, each health professional manages both the patients’ diabetes and CKD, with additional input from a health professional from the other specialty if required, and all patients are discussed at an end of clinic audit. An education program for staff was held prior to initiation of the service.
6. The diabetes-kidney service communicates with the patient’s primary care home team/GP through–i) the same diabetes-kidney care plan that is given to a patient is sent to his/her GP via facsimile on the same day as the consultation ii) a dedicated phone advice service is available to allow GPs to in the event the GP requires clarification of treatment decisions or to seek advice on the management of acute issues.

The model of care (Diabetes Kidney Service) was implemented in 2016 at Monash Health, one of Australia’s largest health service located across Melbourne’s south-eastern suburbs.

Previous longitudinal and pre- and post-design audit studies have reported that combined diabetes and kidney service (similar to the one studied here) may improve clinical target attainment such as HbA1c [14, 15] and enhance patients’ capacity to self-manage their diabetes [16, 17]. Additionally, these studies have suggested that such clinics may attenuate kidney function decline [14, 18, 19]. None of these studies have qualitatively explored the effect of combined services on both attending patients and health-care providers.

The objective of this study was to explore the experiences of patients and health-care providers, with a person-centred, integrated diabetes and kidney service located at Monash Health in the south eastern suburbs of Melbourne.

## Materials and methods

This qualitative study was underpinned by a pragmatic approach [20] and its design framework was guided by grounded theory [21]. Despite the limitations of using grounded theory with focus groups for data collection, the method was appropriate in this hard to reach population where we allowed themes to emerge in order to capture the participants’ health care experiences [22]. We utilised focus groups amongst patients attending the service to explore their experiences and perspectives and triangulated findings with focus groups and semi-structured interviews of health-care providers from the service and primary health-care professionals referring to the service [23]. The study was approved by Monash Health and Monash University Human Research Ethics Committees.

### Participant selection

Patients with diabetes and CKD (stages 3–5, eGFR < 60 mL/min/1.73 m^2^) attending the Diabetes Kidney Service were sampled purposively to ensure a diverse range of experiences was captured. Maximal variation sampling ensured adequate representation of both genders. Separate focus groups were facilitated with participants according to their CKD disease progression (stages 3, 4 and 5) because patients’ experiences are likely to differ according to their CKD stage [11, 23]. All clinical staff from the service, and primary health professionals referring patients to the service (purposively sampled for information rich cases) were also recruited for two separate focus groups. Again, maximal variation sampling ensured adequate representation of both genders in the primary health-care focus group. As some of the primary health-care professionals could not logistically be involved in the focus groups, they participated in a separate semi-structured interview. All participants gave written consent, and patients were ensured that their involvement would not affect their normal medical treatment.

### Data collection and analysis

Discussion questions used for the focus groups and semi-structured interviews (S1 Table) were based on previous questions used in two prior qualitative studies concerning health-care of patients with diabetes and CKD [10, 11]. They were developed in consultation with one Endocrinologist and one Renal Nurse. The discussion were facilitated by the same experienced qualitative researcher (TR). Discussions were audiotaped verbatim, with another facilitator noting behavioural interactions. The de-identified audiotaped discussions were transcribed verbatim and analysed independently by two researchers (CL and EZ) using a generic inductive thematic approach [24, 25]. After immersing themselves in the data by reading the transcript several times, primary patterns within the data were identified and coded into themes in a constant comparative manner. Consensus of the emerging themes was then reached between the three researchers (CL, EZ and TR).

## Results

We conducted five focus groups with patients (CKD stages 3 to 5), specialist health professionals working within the Diabetes Kidney Service and primary care professionals. Additionally we performed two semi-structured interviews with primary care professionals who were not included in the focus group (Table 1). The mean [SD] ages for patients, specialist health professionals and primary care professionals who participated were 68 [5], 45 [11], and 44 [15] years with men being 76%, 50% and 80% of the participant populations respectively.

**Table 1:** Demographic and professional roles of clinic and community participants.

*Clinic participants (N = 6)*	N (Percentage)
Demographics	
Male	3 (50)
Mean age ± SD	45± 11 years
Roles	
Nurse practitioners	2 (33)
Endocrinologist	1 (17)
Nephrologist	1 (17)
Renal nurse	1 (17)
Dietitian	1 (17)
*Primary care professionals* (N = 5)	
Demographics	
Male	4 (80)
Mean age ± SD	44 ± 15 years
*Patients* (N = 21)	
Demographics	
Male	16 (76)
Mean age ± SD Chronic kidney disease stage	68 ± 5 years

3	6 (29)
4	9 (43)
5	6 (29)

Six descriptive themes emerged (Table 2). Three themes were related to the strengths of the Diabetes Kidney Service and these were improved access to services, better integration of care and a perception of improved health and management of health. Three themes were related to areas for improvement and these were improving the process of care, health professional education and patient self-management.

**Table 2:** Illustrative quotes for themes.

Themes	Strengths of the service
Improved access to services	“The–yeah the whole processing of the referral where we refer and then the patient gets in, he’s seen and then feedback is given back. So that timeframe has reduced drastically” Primary care professional 3.
Better integration and continuity of care	“…but because there's a variety of experts, they've all got different fields, actually; some are more diabetes, some are more kidney, and whatever else. And I think that is important, that—because they're interlinked with one another…” CKD 3 patient 24.
Perception of improved health and management of health	“The feedback from the patient was good. She seemed to be well taken care of. Perhaps this is a touch different I think” Primary care professional 1.
	**Areas for improvement**
Process of care	“Yeah. If there was some way that they could maybe, I don’t know, shorten that–that waiting time or to give an individual–oh it’s difficult I know to give everybody individual times” CKD 4 patient 15.
Health professional education	“And just a last point on team education, we did have, early on, some idea about doing regular team, sort of, education sessions and I think—I think that’d be worthwhile to pursue, you know, sort of like a diabetes update, or maybe a renal update, maybe you know, once every six months or something” Specialist health professional 5.
Patient self-management	“Yeah because every time you consult a kidney doctor or a diabetic doctor, they only concentrate on that curative method not on the preventive one” CKD 4 patient 12.

### Strengths of the service

#### Improved access to services

Some primary care professionals who refer patients to the Diabetes Kidney Service felt that referrals for new patients were triaged and processed in a timely way compared to other individual specialist services.

“*The–yeah the whole processing of the referral where we refer and then the patient gets in*, *he’s seen and then feedback is given back*. *So that timeframe has reduced drastically” Primary care professional 3*.

“*I had the same idea with the referral*. *Before it was quite hard but now it is–the referral process is very good*. *There’s some patients that I–that I think that it needs to be followed up by the diabetic clinic” Primary care professional 4*.

Additionally, one primary care professional felt that the referral process was simplified and the Diabetes Kidney Service staff were always available to facilitate this.

“*The access to the clinic was pretty easy*. *She told me exactly where to send it and what to put on there as far as which consultant to name and etc*. *And she got an appointment within 2 to 3 weeks” Primary care professional 1*.

There was consensus among all participants that the Diabetes Kidney Service resulted in fewer appointments for patients who were already faced with the possibility of attending multiple clinics due to their comorbid conditions.

“*I think for the patients they’ve got at least one less appointment” Specialist health professional 4*.

“*…in Dandenong*, *I’ll be so happy*. *So*, *they put both together and they made it the kidney and diabetic clinic for me so that I don’t have to go to two places*. *You know*, *it’s always appointments*, *appointments” CKD 4 patient 11*.

#### Better integration and continuity of care

Patients were confident that having staff from two specialities working together improved the quality and integration of care.

“*…but because there's a variety of experts*, *they've all got different fields*, *actually; some are more diabetes*, *some are more kidney*, *and whatever else*. *And I think that is important*, *that—because they're interlinked with one another…” CKD 3 patient 24*.

Specialist health professionals working at the Diabetes Kidney Service also identified better integration of care as a key strength of the service.

“*I think the aim of trying to address multiple comorbidities is also a very good aim*, *so I think that’s also a strength of the service*. *And having the interaction with our diabetes colleagues*, *from my perspective has been good in terms of learning more about diabetes management*, *so it’s been good for that*, *for my own personal learning*, *to learn more about diabetes and the diabetes service and how that works*. *So*, *there’s quite a few strengths” Specialist health professional 5*.

Participants thought that the processes of the Diabetes Kidney Service resulted in better communication between specialist health professionals and primary care professionals. In particular, the process of sending each patients’ Diabetes-Kidney care plan to primary care after each appointment was notably effective.

“*The feedback that we get is quite detailed*. *There is a general particular template that they follow” Primary care professional 3*.

“*I’ve got 5 to 6 patients and I haven’t had any bad feedback*. *The notes are actually pretty good” Primary care professional 2*.

A majority of specialist health staff working within the Diabetes Kidney Service identified continuity of care as one of the key strengths of the service. Some patients viewed continuity of care as being seen by the same health professional every time they present for clinic and they felt that they were not being seen regularly by the health professionals they were comfortable with.

“*I like the continuity of the patients*, *because you get to know them and it makes for an easier consult*, *and I think it’s nicer for them” Specialist health professional 7*.

“*I want to say that why don’t we have the same specialist every time we come to clinic*? *Why do they keep on changing*?*” CKD 3 patient 17*.

#### Perception of improved health and management of health

A number of patients felt that they were enjoying better health and were able to manage their health better due to attending the Diabetes Kidney Service.

“*So I have to control all that*. *And by doing all this*, *I think I can achieve*. *And the support*, *what you get from the centre*, *the centre here*, *I think is fantastic” CKD 3 patient 23*.

“*Yes*. *Yeah*. *I feel—I feel coming here doing my blood test before—the week before when I come here*, *that's normal*, *14*, *anyway*, *that they keep me on track and*, *if something should be going off track*, *they will warn me*, *and that's important to me” CKD 3 patient 24*.

One primary care professional noted an improvement in their patients’ health which they attributed to the Diabetes Kidney Service.

“*The feedback from the patient was good*. *She seemed to be well taken care of*. *Perhaps this is a touch different I think” Primary care professional 1*.

### Areas for improvement

#### Process of care

All participants generally agreed that the process of care for the Diabetes Kidney Service needed to be improved. Patients felt that they were spending a lot of time in the waiting room before being seen.

“*Yeah*. *If there was some way that they could maybe*, *I don’t know*, *shorten that–that waiting time or to give an individual–oh it’s difficult I know to give everybody individual times” CKD 4 patient 15*.

Additionally, patients reported prolonged consultations due to interruptions that occurred during their review.

“*The service here is good*, *but sometimes I find—after waiting a couple of hours to see the specialist*, *I find that they're interrupted a lot by other people coming in to find out—to ask a question…” CKD 3 patient 19*.

Specialist health professionals were also concerned by these interruptions and they understood that this could have a negative impact on the process of care within the service. However, they accepted that this practice was the nature of an integrated clinic.

“*My constant concern that I’m always nagging the consultants but I understand that that’s part of the role and*, *and I don’t know how that impacts on the patients as well” Specialist health professional 7*.

Both patients and specialist health professionals highlighted that the service had limited physical space. This led to the waiting room being crowded at times.

“*The aesthetics of the room; probably can't do anything about that*, *but that little area does get very*, *very crowded around the 10 o'clock time*, *and so forth” CKD 3 patient 20*.

Patients, primary care professionals and specialist health professionals suggested that the service could be improved by integrating with other specialties, decentralising the service and increasing the number of staff members who work within the service.

“*But the thing that I find really frustrating is that all of–well many of these diabetic people who have kidney disease also have cardiac disease and you don’t have a triple clinic” Primary care professional 5*.

It was noted that if the number of staff members working within the service is increased, this would need to be matched with an increase in physical space. To help manage the currently available space, one specialist health professional suggested increasing the support given to primary care.

“*And that’s perhaps where we could build the capacity with the primary care in terms of being a liaison type*, *kind of like what we’re doing with that travelling person*. *We could sort of*, *provide that peripheral support*, *which is keeping people in their primary care setting” Specialist health professional 7*.

In addition, some health professionals identified a need to streamline the referral criteria so that only patients who can benefit from the service are referred to the service.

“*And having*, *just some clearer referrals so that some of those patient who perhaps could be seen*, *are not being missed” Specialist health professional 3*.

#### Health professional education

Primary care professionals wanted more renal education specifically on guidelines, when to make a referral to a nephrologist and dialysis. The specialist health professionals within the Diabetes Kidney Service reflected on the success of an education session they had a few years back and thought that this would be beneficial to current and new staff to the service.

“*I find that the diabetes side of things is very well dealt with in educational sessions for doctors…*. *But we have very little education on the renal side of things*. *There’s hardly ever a topic to do with the kidney” Primary care professional 5*.

“*And just a last point on team education*, *we did have*, *early on*, *some idea about doing regular team*, *sort of*, *education sessions and I think—I think that’d be worthwhile to pursue*, *you know*, *sort of like a diabetes update*, *or maybe a renal update*, *maybe you know*, *once every six months or something” Specialist health professional 5*.

#### Patient self-management

Most patients reported that the education and health support they received enhanced their self-management. Some patients thought that current self-management support was adequate and that there was an opportunity to reinforcing their current knowledge.

*“…because I said it before*, *it's important to have that six-monthly or four-monthly*, *whatever it may be*, *reinforcement of*, *"Yeah*, *you're on the right track" or*, *"You're not dying tomorrow" or whatever…” CKD 3 patient 24*.

However, others found the education repetitive, with some patients taking the initiative to find their own patient education material.

“*Yes*, *so it’s repeating the whole thing or what I know” CKD 4 patient 11*.

Still other patients found the education material inadequate, and wanted more patient education.

“*Yeah because every time you consult a kidney doctor or a diabetic doctor*, *they only concentrate on that curative method not on the preventive one” CKD 4 patient 12*.

“*Can they give us a diet for the kidneys to improve it*? *Yeah diet for the kidneys*? *Not to improve it*, *just to–yeah to keep it stable” CKD 4 patient 10*.

On the other hand, some health professionals reported that certain patients took longer to process information and needed more support in self-management. This variation in experiences exemplifies the need for patient education and the level of self-management support to be tailored and individualised to the patient. This point was highlighted by both patients and health professionals.

“*I don't know how they could possibly—whether there's a flag on the software that says*, *"Look*, *this person probably understands quite a bit and just is too lazy to do it" as opposed to*, *"This person's new to diabetes and hasn't got the knowledge" and then it may make their time more efficient” CKD 3 patient 20*.

“…*all the patients seeing so many practitioners or dieticians in the one session*, *and then they can be somewhat overwhelmed*, *so they may be a bit disgruntled with the information or the education that they’re given due to the memory and their…ability to recall*, *and I’m wondering*, *there seems to be some patients who are really organised with their bringing in a folder of heir glucose*, *bringing in that folder*, *having what medications they’re on and so on” Specialist health professional 3*.

## Discussion

This qualitative study involving patients with diabetes and CKD, primary care professionals and specialist health professionals identified three key strengths and three areas of improvement for the new integrated diabetes kidney service. The strengths were improved access to services, better integration of care and perception of improved health and management of health. Potential areas for improvement were the process of care, health professional education and patient self-management.

Patients and primary care professionals reported improved access to healthcare through the integrated diabetes and kidney service. The ability of the service to merge two specialist appointments to at least one appointment is likely to improve attendance leading to improved outcomes such as better glycaemic control [26]. The primary care professionals attributed improved access to presence of clear communication from the time of referral up to the time the patient is seen in the Diabetes Kidney Service. However, some primary care professionals were not familiar with the referral criteria highlighting the need for disseminating information about the service to all the primary care professionals within the catchment area of the Diabetes Kidney Service.

All the participants pointed out that the service had resulted in better integration of care. However, primary care professionals thought that the service could be improved further by incorporating other specialties such as cardiology as most of the patients with comorbid diabetes and CKD have cardiovascular diseases. One study reported cost savings when a diabetes and kidney service had cardiology input [27]. However, no patient reported outcomes such as health related quality of life have been reported. An evaluation of the health-related quality of life of patients attending the Diabetes Kidney Service is currently being done.

Perception of improved health and management of health was identified as an important outcome of the Diabetes Kidney Service by both patients and primary care professionals. Patients thought that they were engaged in their care and that necessary investigations were done in the service to monitor their progress. Additionally, primary care professionals received positive feedback about the service from their patients and they also noted that some patients felt better. This could have been due to improvements in clinical outcomes such HbA1c and eGFR as reported previously [28].

Health professional education was highlighted by both primary care professionals and specialist health professionals as an important area for improvement. In this regard, the service may need to address the professional development needs of both primary care professionals and specialist health professionals to promote effective delivery of integrated care. This can be done by implementing rigorous on boarding practices for new clinicians, networking with organisations who have similar integrated models of care and providing learning opportunities to existing personnel through multidisciplinary meetings [29]. It is important, however, to engage both the service users and clinicians in the development and delivery of targeted education programs.

One of the key features of the Diabetes Kidney Service is its ability to incorporate patient self-management education. In this study, some patients preferred the education to be tailored to their needs to avoid having the same information repeated regularly. To effectively address the self-management education needs of patients, the service may need to develop self-management algorithms specific to people with comorbid diabetes and CKD. Up till now, self-management algorithms have been successfully used for patients with type 2 diabetes [30] and these may need to be adapted to suit patients with comorbid diabetes and CKD. Additionally, electronic and mobile education and self-management approaches have been shown to be effective in education and promoting behaviour change in patients with type 2 diabetes [31–33]. This could be extrapolated to and explored in patients with co-morbid diabetes and CKD, to allow individual dose adjustment, reduce cost and health provider burden and address barriers around education and self-empowerment.

The strengths of this study include the rigor in the methodology where two researchers were involved in data synthesis and development of themes. Additionally, the perspectives of all groups of key stake-holders utilising, referring to, or involved in the integrated service were captured, enabling triangulation of data.

Overall, health professionals and patients reported that a co-designed integrated diabetes kidney service improved integration of care and improved health and management of health. However, some aspects of the process of care, health professional education and patient self-management needed improvement highlighting the need to address some patient, health professional and health system barriers to health care.

## Supporting information

S1 Table Patient and health professional interview questions. Interview questions.
(DOCX)
