# Supplementary material for: The impact of an integrated diabetes and kidney service on patients, primary and specialist health professionals in Australia: A qualitative study
Source: PLoS One. 2019 Jul 15;14(7):e0219685. doi: 10.1371/journal.pone.0219685 (PMC6629146; doi:10.1371/journal.pone.0219685)
Supplement: S1 Table — Interview questions. (DOCX) [file pone.0219685.s001.docx]

**Table S1**: Patient and health professional interview questions

| **Patients** | **Health Professionals** |
| --- | --- |
| What are the strengths of the Diabetes Kidney Service (DKS)?  What impact has attending the DKS made to your health?  What are the weaknesses of the DKS?  How easy is it to get the health-care that you need from the DKS (locality, cost, waiting times and parking)?    How could this be improved?  Have you ever missed out a DKS disease appointment if so why?  What do you think about the education provided to help manage your diabetes and kidney disease? How could it be improved?  In your experience, what aspects of the health service could be improved?  Is there anything that we have missed or that you came wanting to say that you haven’t? | Think about the experiences that you have had with the DKS at Monash Health;  What are the strengths of the Diabetes kidney service?  What impact has attending the DKS made to patients’ health?  What are the weaknesses of the DKS?  How accessible do you think current health services are to patients?  What could be done to make health services more accessible to patients (locality, cost, waiting times and parking)?  What do you think about the education provided to help manage patients’ diabetes and kidney disease? How could it be improved?  In your experience, what aspects of the health service could be improved?  Is there anything that we have missed or that you came wanting to say that you haven’t? |
